# Genetic Variants in Cytosolic Phospholipase A2 Associated With Nonsteroidal Anti-Inflammatory Drug–Induced Acute Urticaria/Angioedema

**DOI:** 10.3389/fphar.2021.667824

**Published:** 2021-04-30

**Authors:** Raquel Jurado-Escobar, Inmaculada Doña, José Triano-Cornejo, James R. Perkins, Natalia Pérez-Sánchez, Almudena Testera-Montes, Marina Labella, Joan Bartra, José J. Laguna, Miguel Estravís, José A. G. Agúndez, María J. Torres, José A. Cornejo-García

**Affiliations:** ^1^ Allergy Research Group, Instituto De Investigación Biomédica De Málaga-IBIMA, Malaga, Spain; ^2^ Departamento De Medicina, Universidad De Málaga, Malaga, Spain; ^3^ Allergy Unit, Hospital Regional Universitario De Málaga, Malaga, Spain; ^4^ ARADyAL Network, Instituto De Salud Carlos III, Madrid, Spain; ^5^ Department of Molecular Biology and Biochemistry, University of Malaga, Malaga, Spain; ^6^ CIBER De Enfermedades Raras (CIBERER), ISCIII, Madrid, Spain; ^7^ The Biomedical Research Institute of Malaga (IBIMA), Malaga, Spain; ^8^ Allergy Section, Pneumology Department, Hospital Clinic, Universitat De Barcelona, Barcelona, Spain; ^9^ Allergy Unit, Allergo-Anaesthesia Unit, Hospital Central De La Cruz Roja, Faculty of Medicine, Alfonso X El Sabio University, Madrid, Spain; ^10^ Instituto De Investigación Biomédica De Salamanca (IBSAL), Salamanca, Spain; ^11^ Institute of Molecular Pathology Biomarkers, UEx, Cáceres, Spain; ^12^ Nanostructures for Diagnosing and Treatment of Allergic Diseases Laboratory, Andalusian Center for Nanomedicine and Biotechnology-BIONAND, Malaga, Spain

**Keywords:** NSAID cross-hypersensitivity, urticaria/angioedema, cytosolic phospholipase A2, polymorphisms, arachidomic acid

## Abstract

Nonsteroidal anti-inflammatory drugs (NSAIDs) are among the main triggers of drug hypersensitivity reactions, probably due to their high consumption worldwide. The most frequent type of NSAID hypersensitivity is NSAID cross-hypersensitivity, in which patients react to NSAIDs from different chemical groups in the absence of a specific immunological response. The underlying mechanism of NSAID cross-hypersensitivity has been linked to cyclooxygenase (COX)-1 inhibition causing an imbalance in the arachidonic acid pathway. Despite NSAID-induced acute urticaria/angioedema (NIUA) being the most frequent clinical phenotype, most studies have focused on NSAID-exacerbated respiratory disease. As NSAID cross-hypersensitivity reactions are idiosyncratic, only appearing in some subjects, it is believed that individual susceptibility is under the influence of genetic factors. Although associations with polymorphisms in genes from the AA pathway have been described, no previous study has evaluated the potential role of cytosolic phospholipase A2 (cPLA2) variants. This enzyme catalyzes the initial hydrolysis of membrane phospholipids to release AA, which can be subsequently metabolized into eicosanoids. Here, we analyzed for the first time the overall genetic variation in the cPLA2 gene (PLA2G4A) in NIUA patients. For this purpose, a set of tagging single nucleotide polymorphisms (tagSNPs) in PLA2G4A were selected using data from Europeans subjects in the 1,000 Genomes Project, and genotyped with the iPlex Sequenom MassArray technology. Two independent populations, each comprising NIUA patients and NSAID-tolerant controls, were recruited in Spain, for the purposes of discovery and replication, comprising a total of 1,128 individuals. Fifty-eight tagSNPs were successfully genotyped in the discovery cohort, of which four were significantly associated with NIUA after Bonferroni correction (rs2049963, rs2064471, rs12088010, and rs12746200). These polymorphisms were then genotyped in the replication cohort: rs2049963 was associated with increased risk for NIUA after Bonferroni correction under the dominant and additive models, whereas rs12088010 and rs12746200 were protective under these two inheritance models. Our results suggest a role for PLA2G4A polymorphisms in NIUA. However, further studies are required to replicate our findings, elucidate the mechanistic role, and evaluate the participation of PLA2G4A variants in other phenotypes induced by NSAID cross-hypersensitivity.

## Introduction

Nonsteroidal anti-inflammatory drugs (NSAIDs) are among the most highly consumed medicines worldwide because of their efficiency in the treatment of pain and different inflammatory conditions (Fosbøl et al., 2008; Conaghan, 2012; Duong et al., 2014). The wide usage of NSAIDs is reflected in recent studies reporting that the 57.8% of the Danish (Fosbøl et al., 2008) and the 43.6% of the French (Duong et al., 2014) general populations filled at least one prescription for NSAIDs in the periods 1997–2005 and 2009–2010, respectively; for children, an European study reported that 16.9% have taken at least one NSAID (Valkhoff et al., 2013). However, NSAIDs also trigger the 21–25% of all adverse drug reactions, which includes hypersensitivity to drugs (Kowalski et al., 2011). The most common type of NSAID-hypersensitivity is cross-hypersensitivity, with patients suffering different reactions to chemically unrelated NSAIDs (Kowalski et al., 2011; Kowalski et al., 2013). NSAID-induced acute urticaria/angioedema (NIUA), a phenotype characterized by wheals and/or angioedema development in patients without chronic spontaneous urticaria (CSU), is the most frequent clinical entity (Dona et al., 2012; Kowalski et al., 2013; Dona et al., 2014; Doña et al., 2020).

NSAID cross-hypersensitivity reactions are not mediated by an immunological mechanism but associated with the pharmacological inhibition of cyclooxygenase-1 (COX-1) and increased of cysteinyl-leukotriene (CysLT) levels. COX-1 and the inducible isozyme COX-2 catalyze the initial steps in the biosynthesis of prostaglandins (PGs), leading to the formation of PGG2 and PGH2, which are subsequently transformed into PGE2, prostacyclins, or thromboxanes. COX-1 inhibition blocks PG synthesis, leading to an increased transformation of arachidonic acid (AA) into LTA4, which is metabolized to LTC4 and LTE4 (Doña et al., 2020). Thus, COX-1 inhibition by NSAIDs shunts the AA pathway from PG toward CysLT synthesis, eliciting a reaction in susceptible individuals (Doña et al., 2020).

The COX-1 inhibition hypothesis, first proposed for NSAID-exacerbated respiratory disease (NERD) (Szczeklik et al., 1975), has been supported by different studies showing increased LTE4 levels in urine (Christie et al., 1991; Sladek and Szczeklik, 1993), and nasal (Sladek and Szczeklik, 1993) and bronchoalveolar lavages (Sladek et al., 1994) after a positive salicylic acid (ASA) drug provocation test (ASA-DPT). Moreover, increased baseline levels of LTE4 have been reported in NERD patients (Oosaki et al., 1997; Mita et al., 2001; Higashi et al., 2004; Micheletto et al., 2006) and are associated with the severity of the respiratory reaction during ASA-DPT (Daffern et al., 1999). The COX-1 inhibition hypothesis has been further extended to NSAID-exacerbated cutaneous disease (NECD) (Zembowicz et al., 2003; Setkowicz et al., 2009) and recently to NIUA (Doña et al., 2019).

NSAID cross-hypersensitivity susceptibility is thought to be influenced by genetic factors, as has been suggested by familial aggregation; nevertheless, the inheritance pattern does not seem to be Mendelian (Mastalerz et al., 2006; Caimmi et al., 2014). Despite NIUA being the most frequent clinical phenotype induced by drug hypersensitivity, most genetic association studies have focused on NERD (Jurado-Escobar et al., 2017). Most of these studies have focused centered on a limited number of single-nucleotide polymorphisms (SNPs), without evaluating the overall variability of genes of potential interest. In addition, most studies reporting genetic associations did not include a second population for replication (Oussalah et al., 2016; Jurado-Escobar et al., 2017).

Some genetic associations have been found for variants in enzymes and receptors from the AA pathway and cross-hypersensitivity to NSAIDs (Jurado-Escobar et al., 2017). However, no study has been made of the potential role of study of cytosolic phospholipase A2 (*cPLA2* or *PLA2G4A*) overall genetic variability in NSAID cross-hypersensitivity. This gene encodes a member of the cytosolic phospholipase A2 group IV family, responsible for the hydrolysis of membrane phospholipids to release AA, which is subsequently metabolized to produce eicosanoids, including PGs and CysLTs. However, in a previous study from our group evaluating 9 SNPs across 5 genes, a variant on *PLA2G4A* was found to be associated with NIUA (Ayuso et al., 2015). Moreover, from the 8 variants on chromosome 1, we found to be marginally associated with NIUA in a GWAS, none was related with *PLA2G4A* (Cornejo-García et al., 2013).

In this study, we have evaluated the overall variability of the *PLA2G4A* gene and the potential role of its variants in the development of NIUA. For this purpose, we have selected a set of tagging SNPs (tagSNPs), which encompass the overall variability of this gene, and genotyped a group of NIUA patients and NSAID-tolerant individuals. Moreover, we have included a second unrelated population of NIUA patients and controls to further validate our initial findings. As far as we know, this is the first time such an approach has been performed in cross-hypersensitivity to NSAIDs. Our findings help to disentangle the molecular mechanisms underlying this pathology and to identify potential biomarkers for diagnosis.

## Methods

### Subjects

In this case–control study, two consecutively recruited, independent groups of unrelated cases and controls, considered discovery and replication populations, were included. All patients were aged 18–60 years and self-reported Spanish ancestry. The discovery population was recruited from June 2016 to December 2017 in the Allergy Unit of the Malaga Regional University Hospital (Spain), and consisted on of 269 NIUA patients and 299 NSAID-tolerant controls. The replication population was recruited from January 2018 to December 2019 in the Allergy Units of the Cruz Roja Central Hospital (Madrid, Spain) and Clinic Hospital (Barcelona, Spain), and included 181 NIUA patients and 379 controls (Table 1).

**TABLE 1:** Demographic and clinical data for NIUA patients and NSAID-tolerant controls.

	Discovery population			Replication population		
Variable	NIUA (*n* = 269)	Controls (*n* = 299)	p-value	NIUA (*n* = 181)	Controls (*n* = 379)	*p*-value
Age, years (mean ± SD)	41.1 ± 15	42.6 ± 13.6	0.191	41.7 ± 15	42.7 ± 11.6	0.339
Female, *n* (%)	148 (55)	181 (60.7)	0.169	95 (52.5)	201 (53.0)	0.904
No. of reactions (mean ± SD)^a^	3.8 ± 1.7	NA	NA	3.8 ± 1.0	NA	-
Pharmacological culprit drug, no. of episodes (%)^b^		NA	-		NA	-
*Propionic acid derivatives*	434 (41.9)			276 (41.1)		
*Acetylsalicylic acid*	208 (20.1)			144 (21.5)		
*Pyrazolones*	202 (19.5)			137 (20.4)		
*Arylacetic acid derivatives*	98 (9.5)			62 (9.2)		
*Others*	95 (9.0)			52 (7.7)		

Abbreviation: NIUA, NSAIDs-induced acute urticaria/angioedema.

a No significant differences were found regarding the number of reactions between NIUA patients from the discovery and replication populations (p = 0.755).

b No significant differences were found regarding the culprit pharmacological groups involved in the reactions between the discovery and the replication NIUA patient populations (p = 0.715).

To be included, patients had to have reported at least two episodes of acute urticaria (NIUA) induced by NSAIDs belonging to at least two different chemical groups. Patients with CSU (i.e., the reoccurrence of urticaria for more than 6 weeks with no external trigger) were ruled out according to their clinical history. NSAID cross-hypersensitivity was confirmed by ASA-DPT, as described (Doña et al., 2018).

Age- and sex-matched individuals were included as control subjects. They all reported taking NSAIDs regularly or on occasion without developing a clinical reaction, and had no history of CSU, drug hypersensitivity, rhinitis and/or asthma, or nasal polyposis.

The study was approved by the Ethics Committee of the participant centers and conducted according to the principles of the Declaration of Helsinki. All participants gave informed consent.

### ASA Drug Provocation Test

ASA-DPT was performed in a single-blind manner, giving placebo capsules at different times on the 1st day, as reported previously (Doña et al., 2018). ASA and placebo were given in opaque capsules prepared by the hospital pharmacy service. Other medications were withheld before testing, according to international guidelines (Nizankowska-Mogilnicka et al., 2007).

On the 2nd day, two doses of ASA were administered orally with an interval of 3 h (50 and 100 mg). If no reaction occurred, two larger doses (250 and 500 mg) were administered on the 3rd day, with a 3-h interval. The procedure was stopped if cutaneous and/or respiratory symptoms or changes in vital signs (cardiac rhythm alterations, decrease in peak expiratory flow, or hypotension) appeared, and symptoms were evaluated and treated (Doña et al., 2018). If no symptoms appeared during these periods, this was followed by a 2-day/8-h course of the therapeutic dose (500 mg) after a gap of 24 h (Doña et al., 2018).

### Genotype Determination

Data from individuals of European ancestry were downloaded from the 1,000 Genome Project website (phase 3) (https://www.internationalgenome.org/data-portal/data-collection/phase-3). The region corresponding to *PLA2G4A* plus 2 kb up and downstream from the gene start and end positions, respectively, were extracted using Tabix (Li, 2011) and VCFtools (Danecek et al., 2011) (Table 2). The resulting file was converted to plink format to be loaded into Haploview (Barrett et al., 2005). Non-biallelic and indel markers were not considered. Common tagSNPs (MAF≥5%) were selected based on linkage disequilibrium with Tagger (de Bakker et al., 2005), using pairwise tagging with an r2 threshold of 0.8.

**TABLE 2:** Selected tagSNPs.

tagSNPs	Position (chr 1)	Alleles (M/m)	tagSNPs	Position (chr 1)	Alleles (M/m)
rs979924	186,828,347	C/T	rs12746200	186,880,054	A/G
rs2891263	186,977,509	C/G	rs12747953	186,864,564	T/G
rs2207213	186,989,310	C/T	rs28749294	186,863,777	T/G
rs1980444	186,881,828	C/T	rs35174355	186,885,413	T/C
rs4650715	186,974,056	A/G	rs35180943	186,833,659	G/A
rs6656909	186,904,098	C/T	rs12567135	186,869,201	A/T
rs7416329	186,875,707	T/A	rs12691467	186,950,543	A/G
rs6687896	186,868,289	C/G	rs12720530	186,893,800	T/A
rs7535459	186,833,431	C/A	rs17591849	186,878,442	G/C
rs10752982	186,883,322	T/A	rs7523149	186,827,959	T/C
rs10752988	186,985,153	T/C	rs12143166	186,890,357	A/G
rs2049963	186,882,084	G/A	rs12404877	186,860,960	G/A
rs2064471	186,959,964	C/T	rs12405420	186,938,388	G/A
rs1473676	186,848,796	C/A	rs61544849	186,830,961	T/G
rs6425050	186,842,179	G/A	rs10489406	186,843,995	G/A
rs7547993	186,870,729	G/A	rs61810972	186,873,427	G/C
rs10911927	186,830,957	G/T	rs61810995	186,914,570	G/T
rs10911946	186,886,074	T/C	rs73053761	186,827,870	G/C
rs11577826	186,889,374	C/T	rs72711885	186,953,263	A/G
rs200068879	186,830,965	T/G	rs74482962	186,897,529	G/A
rs11587539	186,946,045	A/T	rs75393128	186,876,336	T/G
rs12088010	186,889,366	T/C	rs76172461	186,847,570	A/T
rs201961032	186,830,953	G/T	rs76587848	186,849,811	G/T
rs4402086	186,983,277	A/G	rs77897442	186,865,541	G/T
rs12068233	186,983,127	T/C	rs78178583	186,843,860	A/C
rs12070830	186,853,262	A/T	rs112214529	186,827,884	A/G
rs12032535	186,962,614	C/T	rs112568781	186,937,435	A/G
rs12042344	186,889,003	T/C	rs111463595	186,827,715	G/T
rs11810276	186,830,945	G/T	rs114333818	186,856,584	G/A

Abbreviations: M, mayor allele; m, minor allele; tagSNPs, tagging single nucleotide polymorphisms.

Genomic DNA was isolated from peripheral blood using the FlexiGene DNA Kit system (QIAGEN). Genotyping during the discovery phase was performed using the iPlex Sequenom MassArray technology, whereas Taqman probes and qPCR were used for the replication population. To ensure that the different technologies gave identical results, 10 samples from each population were genotyped using both systems, finding 100% agreement.

Assuming a disease prevalence of 0.01, a risk allele frequency of 0.2, and a relative risk of >1.5, the discovery phase of this study was estimated to have 80% power to detect an associated tagSNP with *α* = 0.05 (Purcell et al., 2003).

### Statistical Analysis

The Mann–Whitney U test was used for continuous variables comparisons between groups, and the χ2 test for categorical variables. Hardy–Weinberg equilibrium (HWE) and individual SNP association tests with NIUA and NERD were performed using SNPassoc (Gonzalez et al., 2007), evaluating dominant, recessive, and additive models for all tagSNPs. The latter was performed using logistic regressions to calculate tagSNP effects as odd ratios with 95% confidence intervals. A *p*-value ≤0.05 after Bonferroni correction was considered statistically significant. Bonferroni correction thresholds were 8.62E-4 (0.05/58) for the discovery cohort, and 0.0125 (0.05/4) for the replication one.

## Results

### Demographic and Clinical Data

A total of 1,128 individuals were genotyped, comprising NIUA patients and controls from two unrelated, consecutively recruited populations. Table 1 shows demographic and clinical data from both the discovery and replication populations. No significant differences in sex or age between patients and controls were found in either of the considered groups (Table 1). Mean age for NIUA subjects was 41 ± 15 years and 42.6 ± 13.6 years for controls from the discovery population (*p* = 0.191), whereas in the replication population, mean age for NIUA was 41.7 ± 15 years and 42.7 ± 11.6 years for controls (*p* = 0.033).

According to their clinical history, more than 80% of NIUA patients had suffered at least three reactions of cross-hypersensitivity to NSAIDs; however, final diagnosis was established in all patients on the basis of a positive ASA-DPT, as described (Doña et al., 2018). We did not find a statistically significant difference in the total number of reactions when comparing NIUA patients from both the discovery and the replication populations (*p* = 0.755) (Table 1). In addition, no statistically significant difference was found between NIUA patients from the two populations included for the pharmacological drug groups inducing the reactions (*p* = 0.715) (Table 1). For the two patient groups, propionic derivatives were the most frequent triggers, followed by ASA and pyrazolones (Table 1).

### Genetic Association Study

Fifty-eight tagSNPs were selected and genotyped. As none of them deviated significantly from HWE in the control group, they were all retained for association analyses. The genotyping call rate for all tagSNPS was >99% across all individuals.

Table 3 shows the results of genetic association testing in the discovery population under the dominant, recessive, and additive inheritance models.

**TABLE 3:** Association study with the selected tagSNPs in the discovery population of NIUA patients and NSAID-tolerant controls. Statistically significant associations after Bonferroni correction are indicated in bold.

tagSNPs	Model							
	Dominant			Recessive		Additive		
	OR (95% CI)	*p*-value	Corrected *p*-value	OR (95% CI)	*p*-value	OR (95% CI)	*p*-value	Corrected *p*-value
rs979924	0.97 (0.6–1.58)	0.897		1.67 (0.28–10.1)	0.569	1.01 (0.65–1.57)	0.978	
rs2891263	1.19 (0.84–1.66)	0.327		1.01 (0.42–2.42)	0.321	1.14 (0.85–1.52)	0.394	
rs2207213	0.95 (0.64–1.39)	0.783		1.12 (0.41–3.01)	0.829	0.97 (0.7–1.35)	0.871	
rs1980444	1.16 (0.8–1.67)	0.434		1.44 (0.5–3.93)	0.47	1.16 (0.84–1.59)	0.365	
rs4650715	0.94 (0.52–1.69)	0.825		3.36 (0.35–32.51)	0.258	1.03 (0.61–1.79)	0.91	
rs6656909	1.02 (0.68–1.51)	0.939		0.55 (0.1–3.04)	0.484	0.98 (0.68–1.42)	0.931	
rs7416329	0.93 (0.67–1.29)	0.66		0.79 (0.42–1.48)	0.451	0.92 (0.71–1.19)	0.509	
rs6687896	1.03 (0.72–1.48)	0.859		1.12 (0.46–2.72)	0.89	1.04 (0.76–1.41)	0.817	
rs7535459	0.96 (0.69–1.35)	0.825		1.05 (0.67–1.64)	0.828	1 (0.79–1.26)	0.969	
rs10752982	0.054 (0.3–0.96)	0.032		0.55 (0.05–6.14)	0.621	0.57 (0.33–0.98)	0.036	
rs10752988	0.86 (0.61–1.22)	0.407		1.19 (0.78–1.8)	0.410	0.99 (0.79–1.24)	0.921	
**rs2049963**	**2.06 (1.36–3.13)**	5.5E-4	**0.032**	5.73 (1.24–26.41)	8.9E-3	**2.01 (1.39–2.93)**	1.5E-4	**<0.01**
**rs2064471**	**0.27 (0.14–0.52)**	1.47E-5	**<0.001**	1.4 (0.37–5.28)	0.616	**0.43 (0.26–0.73)**	6.6E-4	**0.038**
rs1473676	1.36 (0.89–1.9)	0.066		0.66 (0.37–1.17)	0.149	1.1 (0.86–1.42)	0.451	
rs6425050	0.95 (0.67–1.36)	0.798		0.74 (0.26–2.1)	0.561	0.94 (0.68–1.28)	0.687	
rs7547993	1.16 (0.83–1.62)	0.381		1.05 (0.53–2.09)	0.882	1.11 (0.85–1.46)	0.445	
rs10911927	1.03 (0.74–1.43)	0.869		1.01 (0.53–1.92)	0.985	1.02 (0.78–1.33)	0.89	
rs10911946	1.1 (0.78–1.54)	0.596		0.87 (0.54–1.4)	0.57	1.01 (0.8–1.28)	0.93	
rs11577826	1.28 (0.92–1.79)	0.146		1.17 (0.65–2.11)	0.592	1.2 (0.93–1.56)	0.168	
rs200068879	1.01 (0.69–1.48)	0.965		1.11 (0.35–3.5)	0.853	1.02 ((0.73–1.42)	0.926	
rs11587539	1.39 (0.99–1.95)	0.056		0.92 (0.57–1.48)	0.718	1.16 (0.91–1.48)	0.235	
**rs12088010**	0.56 (0.39–0.81)	0.002		0.24 (0.05–1.13)	0.041	**0.62 (0.42–0.9)**	8.3E-4	**0.049**
rs201961032	1 (0.68–1.48)	0.998		0.66 (0.16–2.8)	0.571	0.97 (0.68–1.39)	0.888	
rs4402086	1.05 (0.76–1.47)	0.753		1.25 (0.65–2.42)	0.501	1.07 (0.82–1.4)	0.599	
rs12068233	0.89 (0.62–1.29)	0.542		1.31 (0.9–1.9)	0.155	1.06 (0.85–1.32)	0.069	
rs12070830	0.76 (0.5–1.16)	0.201		2.28 (0.84–6.16)	0.094	0.93 (0.66–1.3)	0.659	
rs12032535	0.86 (0.6–1.25)	0.442		1.09 (0.75–1.59)	0.636	0.98 (0.78–1.23)	0.856	
rs12042344	1.29 (0.83–2.03)	0.262		3.36 (0.35–32.51)	0.258	1.32 (0.86–2.01)	0.198	
rs11810276	1.02 (0.73–1.43)	0.909		0.97 (0.63–1.52)	0.907	1 (0.79–1.26)	0.987	
**rs12746200**	**0.46 (0.29–0.72)**	5,00E-04	**0.003**	0.44 (0.08–2.29)	0.307	**0.49 (0.32–0.75)**	5.9E-4	**0.035**
rs12747953	1.52 (1.06–2.19)	0.022		1.57 (0.49–5.01)	0.441	1.48 (1.05–2.02)	0.023	
rs28749294	1.02 (0.73–1.44)	0.887		0.91 (0.59–1.41)	0.676	0.99 (0.78–1.24)	0.09	
rs35174355	0.85 (0.6-1–2)	0.355		0.92 (0.39–2.17)	0.855	0.88 (0.66–1.18)	0.396	
rs35180943	1.06 (0.75–1.48)	0.755		1.03 (0.48–2.24)	0.933	1.04 (0.79–1.38)	0.771	
rs12567135	0.95 (0.68–1.32)	0.766		0.51 (0.29–0.9)	0.017	0.85 (0.66–1.09)	0.19	
rs12691467	0.94 (0.63–1.39)	0.754		1.4 (0.37–5.26)	0.62	0.97 (0.68–1.39)	0.881	
rs12720530	1.02 (0.61–1.7)	0.944		1.11 (0.16–7.95)	0.916	1.01 (0.6–1.71)	0.965	
rs17591849	0.49 (0.27–0.89)	0.015		0.55 (0.05–6.14)	0.621	0.52 (0.3–0.91)	0.018	
rs7523149	1.01 (0.7–1.45)	0.959		0.83 (0.28–2.42)	0.732	0.99 (0.72–1.37)	0.954	
rs12143166	1.18 (0.83–1.67)	0.362		1.02 (0.65–1.6)	0.931	1.09 (0.85–1.39)	0.497	
rs12404877	1.06 (0.75–1.52)	0.726		1.64 (0.69–3.89)	0.261	1.11 (0.83–1.49)	0.493	
rs12405420	1 (0.71–1.4)	0.993		1.03 (0.52–2.05)	0.929	1 (0.77–1.32)	0.978	
rs61544849	0.97 (0.69–1.38)	0.869		0.89 (0.34–2.28)	0.801	0.97 (0.71–1.31)	0.822	
rs10489406	0.92 (0.57–1.48)	0.719		1.11 (0.07–17.86)	0.940	0.92 (0.58–1.47)	0.732	
rs61810972	1.03 (0.74–1.44)	0.859		0.72 (0.44–1.17)	0.178	0.93 (0.73–1.19)	0.584	
rs61810995	1 (0.62–1.62)	0.998		0.55 (0.05–6.14)	0.622	0.98 (0.62–1.71)	0.923	
rs73053761	1.17 (0.78–1.75)	0.456		1.5 (0.51–4.37)	0.458	1.17 (0.83–1.65)	0.382	
rs72711885	1.28 (0.92–1.79)	0.143		0.71 (0.35–1.45)	0.343	1.12 (0.86–1.47)	0.403	
rs74482962	0.88 (0.61–1.28)	0.512		0.46 (0.18–1.23)	0.108	0.84 (0.61–1.15)	0.272	
rs75393128	1.13 (0.72–1.78)	0.589		1.11 (0.07–17.86)	0.940	1.13 (0.73–1.74)	0.589	
rs76172461	1.17 (0.76–1.79)	0.483		0.74 (0.12–4.46)	0.740	1.12 (0.76–1.6)	0.563	
rs76587848	0.62 (0.4–1.26)	0.035		0.37 (0.04–3.56)	0.356	0.63 (0.41–0.96)	0.03	
rs77897442	1.02 (0.61–1.7)	0.944		1.11 (0.16–7.95)	0.916	1.02 (0.64–1.63)	0.929	
rs78178583	0.73 (0.47–1.13)	0.153		0.22 (0.03–1.89)	0.112	0.71 (0.47–1.06)	0.091	
rs112214529	1.1 (0.74–1.83)	0.51		1.5 (0.51–4.37)	0.458	1.16 (0.8–1.68)	0.426	
rs112568781	1.25 (0.83–1.87)	0.292		1.11 (0.22–5.56)	0.896	1.21 (0.83–1.77)	0.314	
rs111463595	1.82 (1.22–2.7)	2.8E-4		1.97 (0.57–6.81)	0.273	1.7 (1.19–2.43)	0.003	
rs114333818	0.98 (0.67–1.42)	0.897		0.86 (0.32–2.34)	0.769	0.97 (0.7–1.34)	0.837	

Abbreviation: NIUA, NSAIDs-induced acute urticaria/angioedema.

The rs20449963 variant was associated with an increased risk of NIUA. This remained statistically significant after Bonferroni multiple test correction under the dominant and additive models (OR = 2.06, 95% CI = 1.36–3.13, corrected p-value = 0.032; and OR = 2.01, 95% CI = 1.39–2.93, corrected *p*-value = 0.009, respectively) (Table 3). MAF for controls was 0.079, and for patients 0.152. The tagSNP rs2064471 was associated with a diminished risk of developing NIUA, and this association also was significant under the dominant and additive model when considering the multiple comparisons performed (OR = 0.27, 95% CI = 0.14–0.52, corrected p-value<0.001; and OR = 0.43, 95% CI = 0.26–0.73, corrected *p*-value = 0.038, respectively) (Table 3). MAF values for controls and patients were 0.082 and 0.026, respectively. In addition, the rs12746200 variant was protective after Bonferroni correction according to both the dominant and additive models (OR = 0.46, 95% CI = 0.29–0.72, corrected p-value = 0.003; and OR = 0.49, 95% CI = 0.32–0.75, corrected *p*-value = 0.035, respectively; MAF for controls = 0.125, MAF for patients = 0.208). Finally, the rs12088010 polymorphism was also associated with a diminished risk of NIUA under the additive inheritance model (OR = 0.62, 95% CI = 0.42–0.9; corrected *p*-value = 0.049; MAF values of 0.094 and of 0.119 for controls and patients, respectively) (Table 3).

Marginal associations were found between NIUA and the variants rs12747953, rs17591849, rs76587848, and rs111463595 under the dominant and additive models; however, these associations did not retain statistical significance after multiple testing correction. In addition, the rs12567135 was also marginally associated with a diminished risk of NIUA under a recessive inheritance model (Table 3).

To validate these initial findings, all four nominally associated tagSNPs found in the NIUA discovery cohort were further genotyped in an independent case–control population (replication cohort), and tested for association after verifying that none of them departed significantly from HWE in the control group.

The rs2049963 variant was associated with a statistically significant increased risk for developing NIUA under the dominant (OR = 1.79, 95% CI = 1.14–2.82; corrected *p*-value = 0.048) and additive models (OR = 1.79, 95% CI = 1.21–2.65; corrected *p*-value = 0.016), although the association of this variant with NIUA when considering the recessive model did not surpass Bonferroni correction (Table 4). The polymorphism rs1288010 showed a protective role under the dominant (OR = 0.57, 95% CI = 0.37–0.87; corrected p-value = 0.032) and additive models (OR = 0.59, 95% CI = 0.4–0.88; corrected *p*-value = 0.028). Similar results were found for the variant rs12746200, which was significantly associated with NIUA under these two inheritance models (OR = 0.51, 95% CI = 0.31–0.83, corrected *p*-value = 0.02 for the dominant model; and OR = 0.56, 95% CI = 0.36–0.87, corrected *p* value = 0.028 fort the additive model). Finally, marginal associations were also detected for the rs2064471 (dominant and recessive models) (Table 4).

**TABLE 4:** Association study in the in the replication population of NIUA patients and NSAID-tolerant controls. Statistically significant associations after Bonferroni correction are indicated in bold.

tagSNPs	Model								
	Dominant			Recessive			Additive		
	OR (95%CI)	*p*-value	Corrected p-value	OR (95%CI)	*p*-value	Corrected *p*-value	OR (95%CI)	*p*-value	Corrected *p*-value
**rs2049963**	1.79 (1.14–2.82)	0.012	**0.048**	4.79 (1.22–18.74)	0.017		**1.79** (**1.21–2.65)**	**0.004**	**0.016**
rs2064471	0.55 (0.2–1)	0.042		6.14 (1.23–30.72)	0.015		0.8 (0.49–1.31)	0.366	
**rs12088010**	0.57 (0.37–0.87)	0.008	**0.032**	0.44 (0.09–2.04)	0.253		0.59 (0.4–0.88)	0.007	**0.028**
**rs12746200**	0.51 (0.31–0.83)	0.005	**0.02**	0.66 (0.13–3.3)	0.6		0.56 (0.36–0.87)	0.007	**0.028**

## Discussion

NSAIDs are widely recognized as most frequently medicines involved in drug hypersensitivity reactions, and NSAID cross-hypersensitivity as the most frequent underlying mechanism. Through the pharmacological inhibition of the COX-1 enzyme, PG production is blocked, and the AA metabolic pathway is shunted toward the biosynthesis of CysLTs. In some individuals, this results in a pathological increase of these mediators, which leads to the elicitation of a hypersensitivity response (Szczeklik et al., 1975; Doña et al., 2019). Susceptibility to develop NSAID hypersensitivity seems to be influenced by the interaction of multiple factors, with most of them still unknown. However, there is thought to be a genetic component. Although there is a lack of familial studies on NSAID hypersensitivity, some data support this idea: the familial aggregation of NIUA has been described, even though a classical Mendelian segregation pattern has not been observed (Mastalerz et al., 2006). In addition, the development of NIUA in twins has been reported recently (Caimmi et al., 2014).

In spite of NIUA being the most common NSAID-hypersensitivity phenotype (Dona et al., 2012; Dona et al., 2014; Doña et al., 2020), most genetic association studies have focused on NERD (Jurado-Escobar et al., 2017). Although they have largely investigated polymorphisms in genes related to AA metabolism, no previous work has considered overall common genetic variability in *PLA2G4A*. This gene encodes a cPLA2 group family member, an upstream enzyme that hydrolyzes membrane phospholipids to release AA, which is subsequently metabolized to produce CysLTs. Thus, such an enzyme may be considered the primary source of inflammatory mediators. In addition to the heterogeneity of their results, which can often not be replicated, most previous genetic studies did not take into account the overall variability in genes of potential interest, and rarely included a second population to support their findings (Jurado-Escobar et al., 2017). Here, we have evaluated the overall genetic variability of *PLA2G4A* and its potential association with NIUA development, through a two-stages approach, validating findings in a second population.

Four intronic variants from 58 initially considered tagSNPs in *PLA2G4A* were associated with NIUA in the discovery population and subsequently genotyped in the replication population. Three of them retained statistical signification after Bonferroni correction. The rs2049963 variant was associated with an increased risk of developing NIUA, whereas rs12088010 and rs12746200 were linked to a diminished risk.

We have previously described rs12746200 as associated with NIUA (Ayuso et al., 2015). Although this study did not evaluate the overall variability in *PLA2G4A* and a second replication case-control group was not included, it provides strong support for the results presented here, suggesting a protective role for rs12746200. This variant was also correlated with the development of familial adenomatous polyposis in the single polymorphism evaluation (Umeno et al., 2010). The rs12746200 polymorphism has been also associated with a diminished risk of myocardial infarction in patients with coronary artery disease (Hartiala et al., 2011), an association which was further replicated and which appears to be modulated by dietary polyunsaturated fatty acids, with minor allele carriers showing a significantly lower *PLA2G4A* gene expression (Hartiala et al., 2012). Although the authors did not check if such downregulation was also accompanied by a decrease in protein levels or the effect on other molecules, it has been reported that a PLAG4A blockade may modify gene expression pattern for a variety of proteins in the asthma-associated response (Whalen et al., 2008).

There is a lack of information regarding the clinical impact of the rs2049963 and rs12088010 intronic variants. However, introns are noncoding DNA sequences located between exons (coding regions) which have been widely recognized as key regulators of gene expression and alternative splicing, as they may act as transcription factor binding sites and also modulate transcription rate, nuclear export, and transcript stability (Shaul, 2017). The development of specific clinical entities may be influenced by intron regulation. In this sense, introns have been linked to cardiovascular (Bose et al., 2017; Wang et al., 2019) and autoimmune diseases (AlFadhli, 2013) and cancer (Neves Filho et al., 2012; Blackburn et al., 2016; Tauziède-Espariat et al., 2017; Garinet et al., 2019). Deep-sequencing technologies have also highlighted the role of intronic mutations in human diseases through different molecular mechanisms (Vaz-Drago et al., 2017; Dufner-Almeida et al., 2019). Non-coding polymorphisms in the AA metabolic pathway have been associated with diabetes (Nejatian et al., 2019) and the clinical response to the CysLT receptor antagonist montelukast (Klotsman et al., 2007). We recently reported that some intronic variants in a guanine nucleotide–binding protein to be associated with NIUA (Blanca et al., 2020); moreover, several polymorphisms in 5-lipoxygenase, a central enzyme in CysLT synthesis, have been associated with NERD (Choi et al., 2004; Kim et al., 2005). We cannot exclude also the possibility that these intronic variants are involved in changes in methylation patterns that could affect gene expression, as it has been also shown for other pathologies (Beltrami et al., 2017; Ozaki et al., 2017; Prunicki et al., 2018; McGuire et al., 2019).

In summary, our results strongly suggest that *PLA2G4A* genetic variants play a role in NIUA development. Particular cPLA2 isozymes have been associated with different pathologies (Runarsson et al., 2007; Niknami et al., 2010), and biological processes affecting platelet, endothelial, and leukocyte eicosanoid generation (Hurley et al., 2011; Kirkby et al., 2015) and immune regulation (Rousseau et al., 2015). However, the mechanisms supporting these associations with NIUA at the molecular level are currently unknown. In addition, we cannot exclude the possibility that the tagSNPs we have found here to be associated with NIUA may not be the causal variants but merely tagging them, and that the causal ones may be functional coding variants. To our knowledge, this is the first study addressing overall common genetic variability in *PLA2G4A* in NSAID cross-hypersensitivity. In addition to functional studies, further research expanding the sample size to cover rare variants and including other populations will provide a better understanding of the genetics underlying NSAID cross-hypersensitivity and its different phenotypes. This will help in the search for potential biomarkers for prognostic and diagnosis purposes.

## Data Availability

The raw data supporting the conclusions of this article will be made available by the authors, without undue reservation.
